# Random Error Reduction Algorithms for MEMS Inertial Sensor Accuracy Improvement—A Review

**DOI:** 10.3390/mi11111021

**Published:** 2020-11-21

**Authors:** Shipeng Han, Zhen Meng, Olatunji Omisore, Toluwanimi Akinyemi, Yuepeng Yan

**Affiliations:** 1 Institute of Microelectronics, Chinese Academy of Sciences, Beijing 100029, China; hanshipeng@ime.ac.cn (S.H.); yanyuepeng@ime.ac.cn (Y.Y.); 2 University of Chinese Academy of Sciences, Beijing 100049, China; 3 Shenzhen Institutes of Advanced Technology, Chinese Academy of Sciences, Shenzhen 518055, China; omisore@siat.ac.cn (O.O.); tolu@siat.ac.cn (T.A.)

**Keywords:** MEMS gyroscope, MEMS accelerometer, random error reduction, signal processing algorithms

## Abstract

Research and industrial studies have indicated that small size, low cost, high precision, and ease of integration are vital features that characterize microelectromechanical systems (MEMS) inertial sensors for mass production and diverse applications. In recent times, sensors like MEMS accelerometers and MEMS gyroscopes have been sought in an increased application range such as medical devices for health care to defense and military weapons. An important limitation of MEMS inertial sensors is repeatedly documented as the ease of being influenced by environmental noise from random sources, along with mechanical and electronic artifacts in the underlying systems, and other random noise. Thus, random error processing is essential for proper elimination of artifact signals and improvement of the accuracy and reliability from such sensors. In this paper, a systematic review is carried out by investigating different random error signal processing models that have been recently developed for MEMS inertial sensor precision improvement. For this purpose, an in-depth literature search was performed on several databases viz., Web of Science, IEEE Xplore, Science Direct, and Association for Computing Machinery Digital Library. Forty-nine representative papers that focused on the processing of signals from MEMS accelerometers, MEMS gyroscopes, and MEMS inertial measuring units, published in journal or conference formats, and indexed on the databases within the last 10 years, were downloaded and carefully reviewed. From this literature overview, 30 mainstream algorithms were extracted and categorized into seven groups, which were analyzed to present the contributions, strengths, and weaknesses of the literature. Additionally, a summary of the models developed in the studies was presented, along with their working principles viz., application domain, and the conclusions made in the studies. Finally, the development trend of MEMS inertial sensor technology and its application prospects were presented.

## 1. Introduction

The microelectromechanical systems (MEMS) inertial sensor is an instrument that is used to measure angular velocity and acceleration [1,2]. In general, MEMS inertial sensors are referred to as MEMS gyroscopes and MEMS accelerometers, and are mainly composed of a micromechanical sensing part, signal processing circuits, and a microprocessor part [3,4,5]. MEMS inertial sensors have many advantages that have earned them varying application areas, but importantly, merits such as the small size, low cost, high precision, ease of integration, and higher possibility of mass production are a major reason that MEMS inertial sensors have become prominent [6,7,8]. Therefore, MEMS inertial sensors are widely popular in civil and military applications. According to the application scenario, in the civilian field, MEMS inertial sensors are mainly used in consumer portables such as mobile phones, game consoles, digital cameras, robots, and medical devices for health care [9,10,11,12,13,14]. In the military field, MEMS inertial sensors are commonly employed in high-end markets such as optoelectronic devices, aerospace, torpedo, missile, rockets, and so on [15,16,17]. However, until now, compared with laser gyroscopes, fiber gyroscopes, mechanical gyroscopes, and accelerometers, the high-end application range of the MEMS inertial sensor is still limited [18,19].

**Figure 1:**
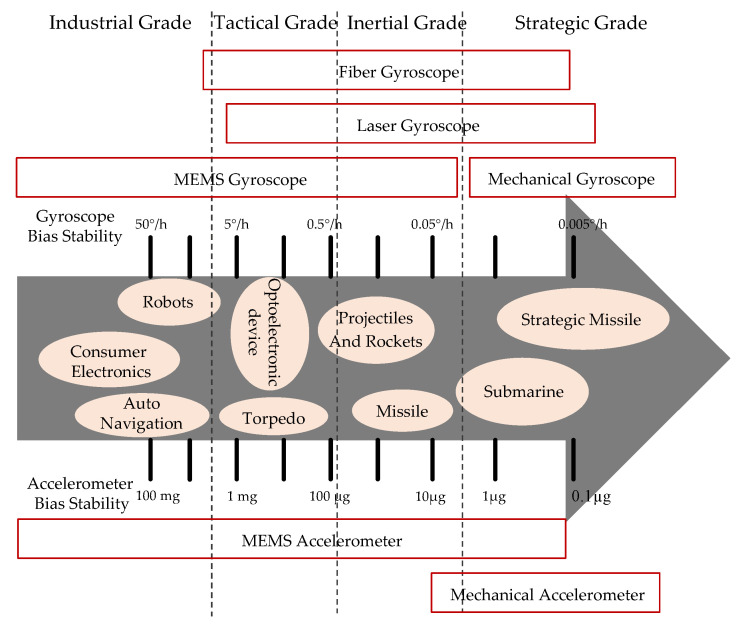
The bias stability of inertial sensors.

A reason for the huge difference between the application fields of the inertial sensors is mainly due to accuracy and price. Bias stability is an important parameter to reflect the accuracy of inertial sensors [8,20]. Therefore, on the basis of the sensor’s precision (i.e., accuracy of the inertial sensors), bias stability is commonly used to represent the inertial sensor’s developmental level, including laser gyroscopes, fiber gyroscopes, mechanical gyroscopes, and accelerometers, especially for MEMS inertial sensors [8,18,21]. As shown in Figure 1, the MEMS inertial sensor can be said to have a wide area of application, ranging from industrial to strategic grade, while the fiber gyroscope, laser gyroscope, mechanical gyroscope, and accelerometer are mainly applied at tactical grade, inertial grade, and strategic grade for their high precision, however their bulkiness and expensive price restrict expansion to the downstream markets, and they are mainly used in high-end military markets at present. Conversely, MEMS inertial sensors are moving towards inertial and strategic high-end applications, due to their low-price advantage and finally, they are likely to achieve a monopoly from the downstream market to the upstream high-end market.

In recent years, with the development of 5G, Internet of Things (IoT), Artificial Intelligence (AI), national defense construction, and some special fields such as deep sea [22], deep space [23], deep drilling [24,25], earthquake monitoring [26], and structural health monitoring [26,27,28], MEMS inertial sensors’ demand is becoming bigger. However, the existing accuracy of MEMS inertial sensors seems insufficient to meet the needs of market applications in those areas. Therefore, improving the accuracy of MEMS inertial sensors is the only way to expand the application range. Presently, the low accuracy of MEMS inertial sensors is the biggest challenge that limits its development. The main reason is that the micro size of the MEMS inertial sensor makes it more vulnerable to environmental changes [29,30]. These uncertain factors can cause various noises, which in turn affect the sensor’s output. In addition, the mechanism of noise generation is complicated, and it is very difficult to quantitatively compensate the error caused.

The error sources of MEMS inertial sensors are mainly from mechanical noise, electronic noise, environmental noise, and other random noise sources [29,31,32]. This kind of uncertain noise or random error limits the accuracy of the sensor and its applicability in different fields. Therefore, this paper mainly presents the existing algorithms that have been developed for processing random error signals obtained from MEMS inertial sensors. As an important part of MEMS inertial sensors, random error reduction algorithms can minimize the error uncertainty from the output signals, and as well, they improve the accuracy and reliability of MEMS inertial sensors.

The purpose of this article is to systematically review the random error signal processing algorithm, including some error reduction methods that have been developed for MEMS inertial sensor output signal processing. The content can provide guidance for users to choose the most suitable MEMS inertial sensor error signal processing algorithm to improve the precision of the MEMS inertial sensor. The remainder of this paper is organized as follows. Section 2 presents the approach of inclusion and exclusion criteria taken for searching and inclusion of the research papers. Section 3 presents the principle of different algorithms used for classifying the signal processing methods. Section 4 discusses all the choosing algorithms. The conclusion of this review paper is presented in Section 5 and also suggests the development direction of MEMS inertial sensor technology.

## 2. Materials and Methods

The review was performed considering related studies published between 2010 and 2020 in journals or conferences that were indexed in four global databases. The selection criteria were carefully designed to consider error signal processing papers, with focus on MEMS accuracy improvement and broad application prospects. For this purpose, a systematic search was conducted on Web of Science, IEEE Xplore, Science Direct, and Association for Computing Machinery Digital Library (ACM). The selected words were set as (MEMS gyroscope; MEMS accelerometer; MEMS inertial sensor; MEMS inertial measurement unit), (noise; drift; error; signal processing), and (reduction, elimination, suppress calibration, compensation, modeling).

A total of 486 articles were initially located and downloaded. Specifically, these included 226 articles from the Web of Science, 115 from IEEE Xplore, 93 from Science Direct, and 52 from ACM. After downloading, articles that were out of the time-frame between 2010 and 2020 were removed (Step 1); thus, 352 articles were retained. Additionally, a total of 96 duplicate articles and all non-English papers were removed, leaving us with 256 articles (Step 2). Furthermore, we removed the papers following step 3 (remove those focusing on MEMS inertial sensor application and non-random error processing algorithms), and 112 articles were kept. Finally, 49 papers were selected as relevant to the topic based on step 4 (remove non-MEMS inertial sensor accuracy improvement papers).

The inclusion criteria were observed by analyzing the title and abstract, focusing on random error reduction algorithms for improving MEMS inertial sensor intrinsic accuracy, without considering the prototype or product application filed. The main exclusion criteria after the steps highlighted above were non-English articles and papers with non-applicable theme such as MEMS inertial sensor raw signal processing. In particular, although the title of some papers were on error signal processing, we found out that they were based on the use of MEMS inertial sensors, and do not pay attention to the improvement of the performance of MEMS inertial sensors, thus such papers were also deleted.

**Figure 2:**
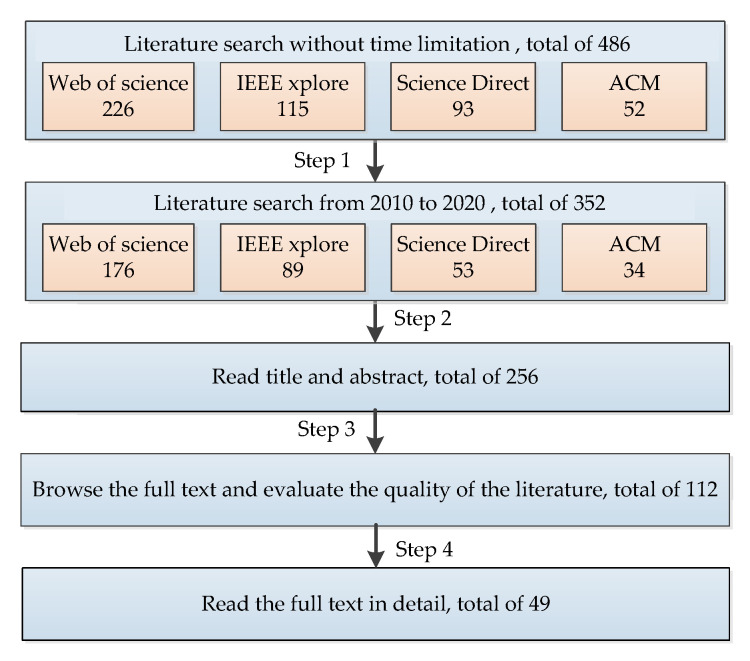
The flow chart of the search strategy.

The main authors read through the titles and abstracts of the search results, and conducted a preliminary analysis to determine whether they match the inclusion criteria. Then, the full texts’ quality was evaluated in detail by the main authors. According to the exclusion criteria, articles not fitting were excluded. Therefore, the final 49 articles left were used for writing this review paper. A flow chart of the search strategy adopted is presented in Figure 2.

## 3. Results

**Figure 3:**
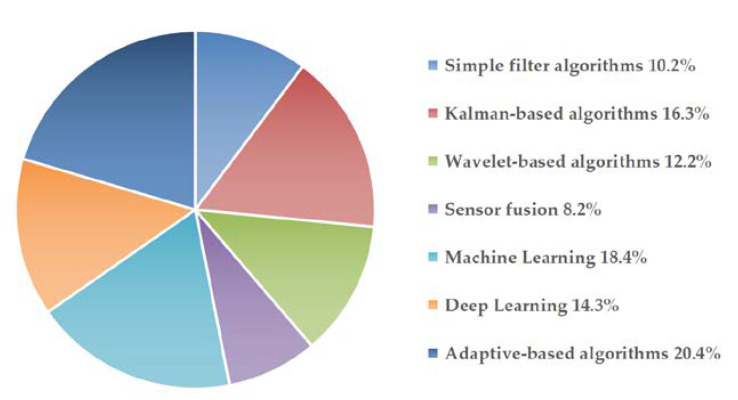
Proportions of the types of algorithms in the reviewed results.

From the 49 reviewed articles, 30 random error signal processing algorithms were summarized, and they were divided into seven groups namely, simple filter algorithms, Kalman-based algorithms, wavelet-based algorithms, sensor fusion algorithms, machine learning algorithms, deep learning algorithms, and adaptive-based algorithms. Some details of these algorithms are presented below. First, it is vital to state that all these algorithms were aimed at reducing raw signal error for MEMS inertial sensor precision improvement. The proportions of the seven types of algorithms in the reviewed results are shown in Figure 3. It can be observed from the pie chart that the review paper had considered a well-balanced ratio of the seven categories of the random error signal processing algorithms.

### 3.1. Simple Filter Algorithms

#### 3.1.1. Fading Memory Filter (FMF)

A low pass filter based on the alpha-beta filter, which is called the fading memory filter (FMF), was used for reducing the amount of noise from the MEMS gyroscope raw data [33]. This type of low pass filter has better characteristics about the computational overhead, the rate of noise reduction, and the phase-delay of the filter. The mathematical mode of this filter was described in [33,34], where $X_{sk}$ is the smoothed signal, T is the sampling time, and $X_{ok}$ is the measured signal. $X_{pk}$ is the predicted signal. $V_{sk}$ is the second derivative of the measured signal, and α and β are filter gains.

(1) $$X_{sk}=X_{pk}+(1-\beta )(X_{ok}-X_{pk})$$

(2) $$X_{sk}=X_{pk}+\alpha (X_{ok}-X_{pk})$$

(3) $$V_{sk}=V_{sk-1}+\frac{\beta }{T}(V_{ok}-V_{sk-1})$$

(4) $$X_{pk}=X_{sk-1}+TV_{sk-1}$$

#### 3.1.2. Morphological Filter (MF)

**Figure 4:**
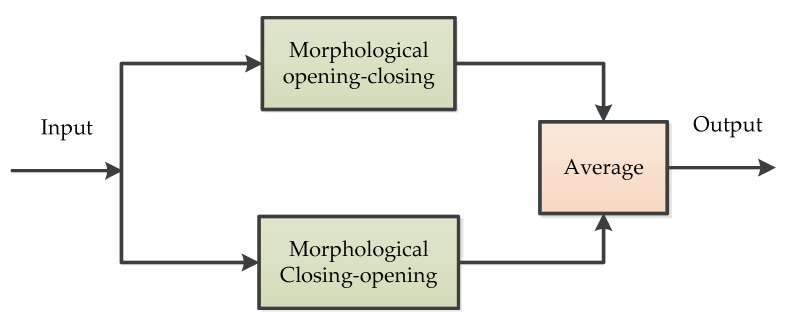
The principle of morphological filter.

MF is a simple low pass filter and a nonlinear time-frequency analysis method capable of extracting local features and eliminating instantaneous impulses [35]. In MF analysis, four basic operators are often used. These include dilation, erosion, opening, and closing operations [36]. The opening and closing operators are established based on the dilation and erosion operators. Opening operation can suppress the positive impulse noise in the raw signal, while closing operation can suppress the negative impulse noise in the signal [36]. If the mixed signal of positive and negative impulse noise need to be filtered out, the MF needs to cascade a combination of opening and closing operations [37]. To overcome the limitation of the MF in handling MEMS gyroscope measurement noise, an improved MF based on variational mode decomposition was proposed for denoising of the raw output signal from the MEMS gyroscope [36,37]. The basic principle of MF is commonly showed in Figure 4 [37].

#### 3.1.3. Moving Average Filter (MAF)

A simple moving average filter (MAF) algorithm is used to suppress the signal noise in the unstable period, and the reason is the sample variance of the current sample can be quite large and exceed a predefined threshold [38]. It is predominantly used to keep steep edge features of the signal. For noise reduction, the MAF is commonly adopted to process the signal unstable periods, hence it can be used in the MEMS gyroscope’s dynamic signal output [38]. Generally, the MAF can be modeled according to equation (Equation (5)), where 2p is the data length of MAF calculation, x(k+i) is the input time series involving 2p sample steps, and *k* is the sampling time.

(5) $$\overset{\wedge }{x}(k)=mean(\sum_{\begin{array}{l} i=-p\\ i\neq 0 \end{array}}^{p}x(k+i))$$

#### 3.1.4. Variable Bandwidth Filter (VBF)

**Figure 5:**
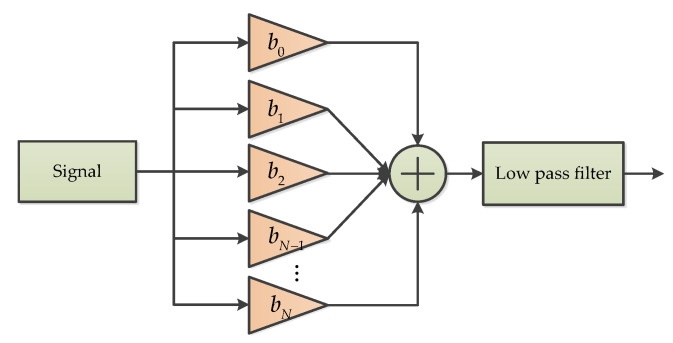
The process of Variable bandwidth filtering.

A variable bandwidth filtering (VBF) method was proposed to suppress the effects of vibration and sensor noise [39]. In this method, the sinusoidal estimation is used to continuously adjust the filtering bandwidth of accelerometer output data to restrain the influence of vibration and noise before attitude estimation is treated [39]. The flow chart of the VBF process is depicted in Figure 5. The proposed filtering process is adaptive because the bandwidth of the entire filtering process can vary with the frequency content of the signal, and it can be divided into two main stages [39]. In the first stage, a variable bandwidth Kaiser windowed filter with a coefficient of $\left[b_{1}b_{2}\cdots \cdots b_{N-1}b_{N}\right]$ was used to filter the signal, while the second stage adopted a low pass filter (LP) with a variable decomposition, which is called an LP wavelet filter [39]. In addition, the coefficients of the Kaiser windowed LP filter can be calculated with a mathematical formula, which was discussed in reference [39].

### 3.2. Kalman-Based Algorithms

#### 3.2.1. Kalman Filter (KF)

**Figure 6:**
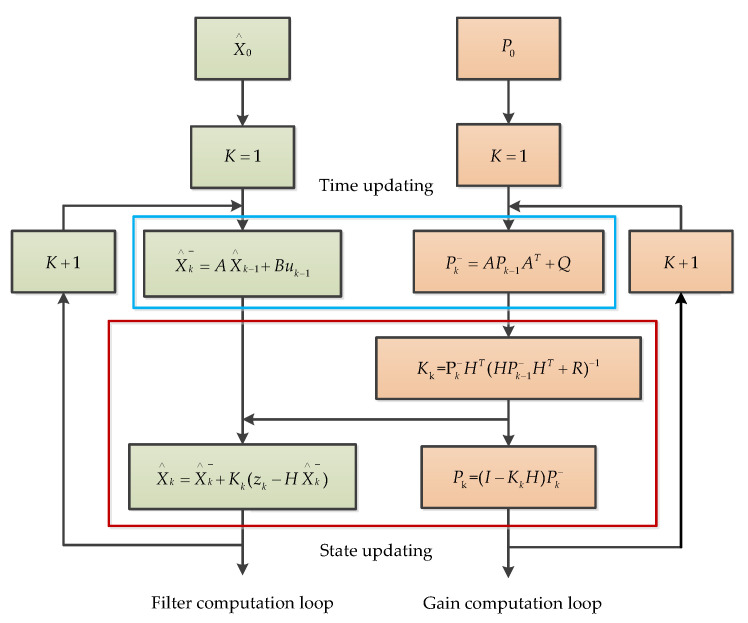
The process of the Kalman filter.

The Kalman filter (KF) is a recursive filter proposed by Kalman for time-varying linear systems [40]. It is an optimal estimation theory and algorithm that can be applied to dynamic systems subject to random interference, such as MEMS gyroscope random drift compensation and temperature drift compensation [41,42]. KF is a common approach, in which recursive algorithms are implemented by a computer program for the purpose of signal state estimation. Each recursive process includes two processes—time update and status update. From the perspective of the calculation process, it includes two loops—a filter computation loop and a gain computation loop. The main structure of KF is presented in Figure 6.

Where $\overset{\wedge }{X}$ is the posteriori estimated state, ${\overset{\wedge }{X}}_{k}^{\_}$ is the priori state, μ is the control vector, z is the measurement signal, k is a discrete point in time, A is the state transition model, B is the control input model, P is the error covariance, Q is the process noise covariance, K is the Kalman gain, H is the measurement matrix, R is the measured noise covariance, and I is the identity matrix.

#### 3.2.2. Extended Kalman Filter (EKF)

The extended Kalman filter (EKF) is derived from the basic KF, and it is one of the most common algorithms for bias drift and noise suppression from the outputs signal of the MEMS gyroscope [43]. The basic idea of EKF is to linearize the nonlinear system and then, carry out KF, while Taylor series is often used to linearize the nonlinear system, so EKF is a kind of pseudo nonlinear KF [43]. The non-linear and linear relations used in EKF are given in Equations (6)–(9), where f and h are all non-linear function, f is posttest of the motion model position, h is the posttest of measurement data, W is the process noise, and V is measurement noise.

Nonlinear:

(6) $$X_{k+1}=f(X_{k})+W_{k}$$

(7) $$Y_{k}=h(X_{k})+V_{k}$$

Linear:

(8) $$F(k+1|k)=\frac{\partial f(X_{k})}{\partial X}|X=X(k|k)$$

(9) $$H(k)=\frac{\partial h(X_{k})}{\partial X}|X=X(k|k-1)$$

#### 3.2.3. Incremental Kalman Filter (IKF)

The KF can estimate the system and measurement noise. However, in the inertial navigation system field, the system error of the measurement equation is commonly unknown, and the model parameters are also uncertain, which may cause the error to become larger and the convergence of the KF to deteriorate [44]. To resolve this problem, an improved IKF algorithms is proposed [44]. The idea of this algorithm is to choose the increment of two continuous measurement values as the measurement value to reduce the system error [44]. Thus, the state equation and the measurement equation of IKF are obtained.

(10) $$X_{k}=\Phi_{k,k-1}X_{k-1}+W_{k-1}$$

(11) $$\Delta Z_{k}=H_{k}X_{k}-H_{k-1}X_{k-1}+V_{k}$$

where $X_{k}$ is the n dimension state vector, $W_{k}$ is the p-dimension state noise variance vector, and $\Phi_{k,k-1}$ is the n×n state transfer matrix of the system. $\Delta Z_{k}$ is the incremental of the m dimension measurement vector, and $\Delta Z_{k}=Z_{k}-Z_{k-1}$, where $Z_{k}$ is the measurement vector. $H_{k}$ is the system observation matrix and $V_{k}$ is the m dimension measurement noise variance vector.

#### 3.2.4. Strong Tracking Kalman Filter (STKF)

The KF algorithm has poor ability to track the state of the MEMS gyroscope, but the STKF can track the state change of the gyroscope very well [42,45]. The STKF introduces a fading factor $\lambda_{k}$ to adjust the KF gain K online, so that the filter residuals are satisfied with the orthogonality principle [45]. The STKF is as follows:

(12) $$X_{k+1}=\phi_{\left(k+1,k\right)}X_{k}+\Gamma_{\left(k+1,k\right)}\omega_{k}$$

(13) $$Y_{k+1}=H_{k+1}X_{k+1}+\nu_{k+1}$$

(14) $$P_{\left(k+1,k\right)}=\lambda_{k}\phi_{\left(k+1,k\right)}P_{k}\phi_{\left(k+1,k\right)}^{T}+Q_{k}$$

where $X_{k+1}$ is the state series of system, $Y_{k+1}$ is the measurement series of system, $P_{\left(k+1,k\right)}$ is the state prediction variance, $\phi_{\left(k+1,k\right)}$ is the state transition matrix, $\Gamma_{\left(k+1,k\right)}$ is the noise input matrix, $\omega_{k}$ is the processing noise, $H_{k+1}$ is the measurement matrix, $\nu_{k+1}$ is the measurement noise, and $Q_{k}$ is the error variance matrix.

#### 3.2.5. Discrete Time Kalman Filter (DTKF)

**Figure 7:**
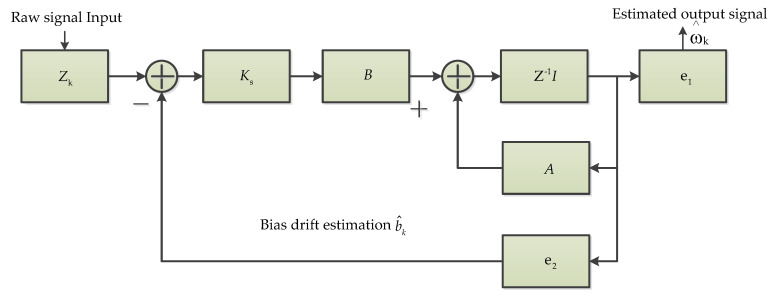
The discrete time Kalman filter for real angular rate signal and bias drift estimation [46].

A Discrete Time Kalman filter (DTKF) was designed by a steady-state filter gain obtained from the analysis of KF observability [46]. In the design of DTKF [46], a system state vector based on true angular velocity ω and offset drift b is used. The steady-state filtering gain $K_{s}$ is analyzed offline by using the basic discrete iterative KF method. I is the identity matrix. Z is the measured angular rate vector as input. The extract vector for ω and b are defined as $e_{1}=[1,0]$ and $e_{2}=[0,1]$, respectively. Depending on the eigenvector matrix S and eigenvalues $\lambda_{1}$ and $\lambda_{2}$, parameters A and B can be calculated. According to these parameters, the DTKF is achieved as showed in Figure 7 [46].

### 3.3. Wavelet-Based Algorithms

#### 3.3.1. Wavelet Threshold (WT)

**Figure 8:**
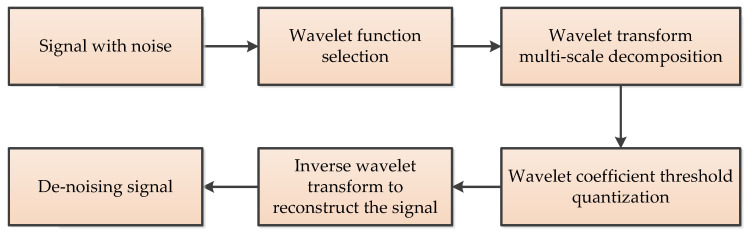
The denoising process of the wavelet threshold.

The wavelet threshold (WT) is commonly used for denoising signals generated in MEMS gyroscope output [47]. Its basic idea is setting a critical threshold value λ for denoising the gyroscope’s output signals. If the wavelet coefficient is less than λ, the coefficient is considered to be mainly caused by noise, and this part of the coefficient is removed. If the wavelet coefficient is greater than λ, it is considered that the coefficient is mainly caused by the signal. This part of the coefficient is kept, and then the inverse wavelet transform is carried out on the processed wavelet coefficient to obtain the denoised signal. WT denoising has two key points: one is the selection of threshold value, the other is the selection of threshold function [47]. The denoising process of wavelet threshold is shown in Figure 8.

#### 3.3.2. Improved Wavelet Threshold (IWT)

The key to WT denoising is to choose a suitable wavelet threshold, commonly used in threshold functions including the hard threshold and soft threshold. The hard threshold and soft threshold are easily achieved in the practical application of engineering [48,49], where $W_{j,k}$ is the wavelet coefficient, ${\overset{\wedge }{W}}_{j,k}$ is the wavelet coefficients after quantization; λ is the threshold.

Hard threshold function:

(15) $${\overset{\wedge }{W}}_{j,k}=\{\begin{array}{c} W_{j,k},\left|W_{j,k}\right|\geq \lambda \\ 0,\left|W_{j,k}\right|\&<\lambda \end{array}$$

Soft threshold function:

(16) $${\overset{\wedge }{W}}_{j,k}=\{\begin{array}{c} W_{j,k}-\lambda ,W_{j,k}\geq \lambda \\ 0,-\lambda \leq W_{j,k}<\lambda \\ W_{j,k}+\lambda ,W_{j,k}<\lambda \end{array}$$

However, both the soft and hard thresholds have certain disadvantages [48,49]. To overcome the shortcoming of hard and soft threshold function, an IWT function is proposed as Equation (17), where n is an adjustable factor, the real number is greater than 0, which can be set following actual engineering requirements.

(17) $${\overset{\wedge }{W}}_{j,k}=(1-e^{-{\left|W_{j,k}/\lambda \right|}^{n}})W_{j,k}$$

#### 3.3.3. Adaptive Stationary Wavelet Threshold (ASWT)

The stationary wavelet threshold (SWT) is also called unsampled wavelet transform [50]. The main feature of SWT is its redundancy and translation invariance, which gives a more approximate estimate. However, it is not appropriate to use the same threshold at each decomposition scale, which will result in the useful signal being eliminated at the low scale, while some noise is retained at the high scale [50]. Therefore, ASWT can be considered to solve this problem as depicted in Equations (18) and (19).

(18) d(n)=x(n)+ζ(n),n=1,⋯,N

(19) $$\lambda =\sigma \sqrt{\left(2\ln N\right)}/\ln(j+2)$$

where x(n) is the signal without noise, d(n) is the original signal, ζ(n) is the noise, σ is the original signal standard deviation, N is the length of signal, j is the decomposition scale, and λ is the adaptive threshold.

#### 3.3.4. EMD-Based Wavelet Threshold (EMD-WT)

**Figure 9:**
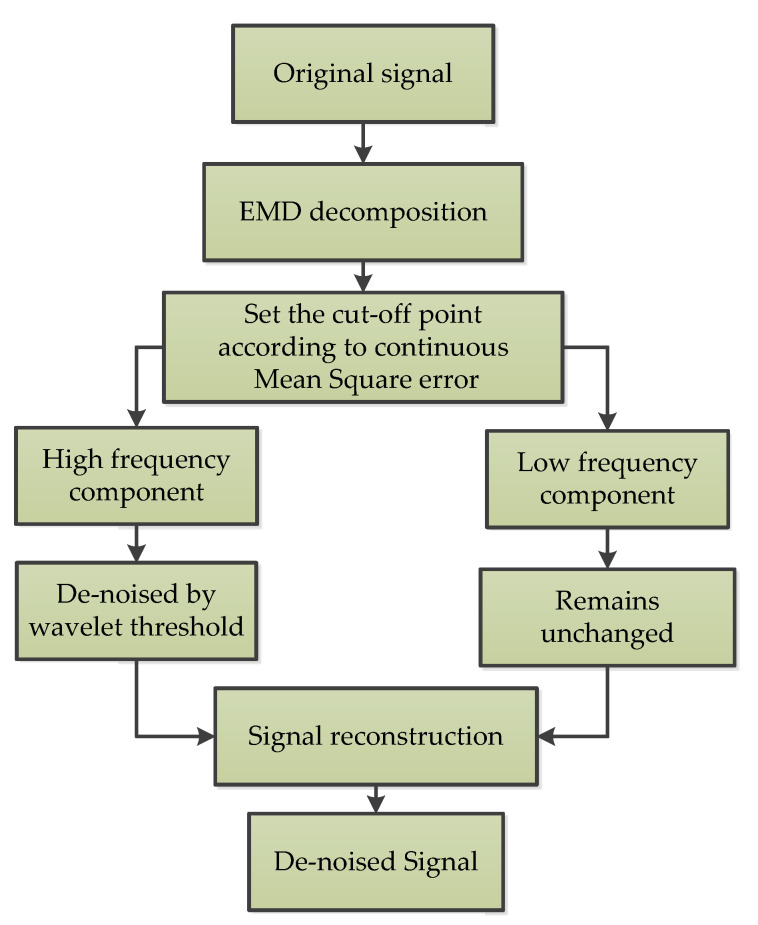
The empirical mode decomposition based wavelet threshold denoising process [51].

The empirical mode decomposition based wavelet threshold (EMD-WT) method, which is the combination of wavelet thresholding and empirical mode decomposition (EMD), is introduced in the paper [51] for MEMS accelerometer signal denoising. Firstly, the output signal is decomposed by EMD to obtain its intrinsic mode function (IMF). Then, the wavelet threshold is used to denoise the high-frequency IMF components, and the low-frequency IMF components remain unchanged. Finally, the denoised high-frequency IMF components is combined with the unprocessed low-frequency IMF components and residuals to achieve signal denoising. The flow chart of this algorithm is shown in Figure 9 [51].

### 3.4. Sensor Fusion Algorithms

#### 3.4.1. Virtual Gyroscope (VG)

**Figure 10:**
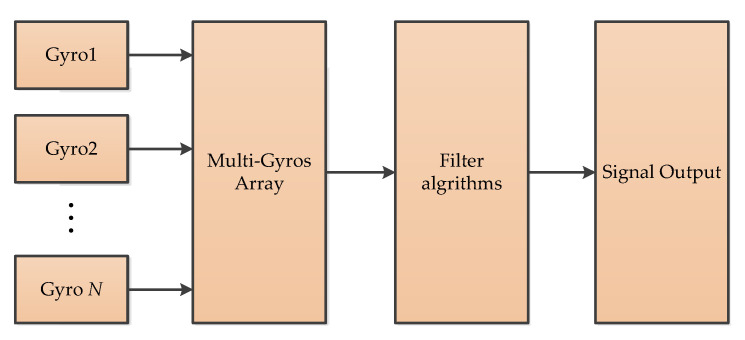
The principle of virtual gyroscope.

The virtual gyroscope (VG) is composed of multiple gyroscopes of the same model and batch [52]. These gyroscopes have the same manufacturing process and materials, and the same data acquisition and processing environment. By designing the appropriate filter algorithm, higher precision measurement can be achieved. In [52,53], they used six gyroscopes’ data to optimize output measurement accuracy, and the principle of VG is as shown in Figure 10.

#### 3.4.2. Heterogeneous Fusion (HF)

**Figure 11:**
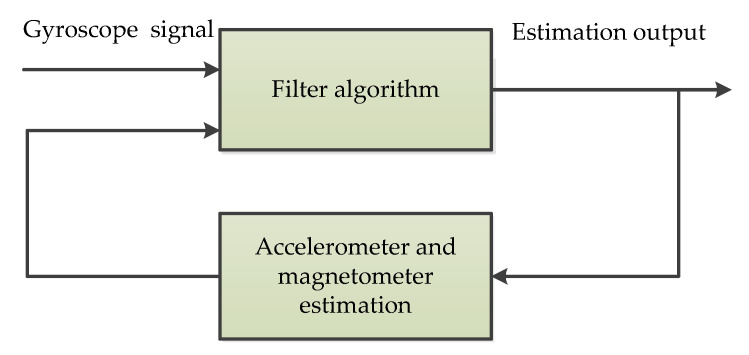
The simplified fusion scheme of heterogeneous fusion.

The heterogeneous fusion (HF) is a type of novel sensor fusion algorithm, which discusses an innovative adaptive fusion algorithm based on the estimation of the mean square error of all variables used in real-time processing [54]. The scheme shown in Figure 11 describes the algorithm that can be used for fusion of the Euler angles computed from gyroscope, accelerometer, and magnetometer readings. The fusion algorithm is based on the concept of compensating the difference between incrementally integrated Euler angles (α-gyro, β-gyro, and γ-gyro) and absolute but noisy Euler angles (α-acc, β-acc, and γ-mag). This method eliminates the need for steady-state detection and offline calibration. Because it can run all the time, the method can continuously compensate for long-term drift when the sensor is in use [54].

#### 3.4.3. Combination Sensors (CS)

**Figure 12:**
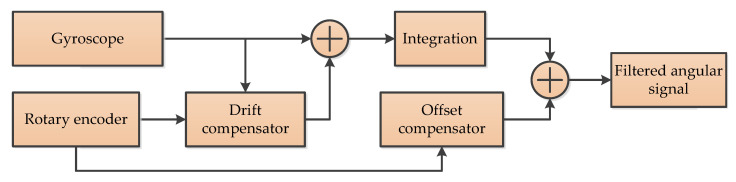
The structure of Drift offset compensator [55].

To avoid noise amplification when estimating angular rates from encoder sensor signals and the drift from the angular velocity sensors—especially for a cheap MEMS gyroscope—a sensor fusion algorithm to angle and angular rate estimation is proposed [55]. Actually, it is the combination of a low-price MEMS gyroscope and low-resolution encoders. This method utilizes the encoder to eliminate the drift of the angular rate signal and integrates the resulting signal to obtain estimates of the angle. The basic concept is illustrated in Figure 12 [55].

### 3.5. Machine Learning

#### 3.5.1. Back Propagation Neural Network (BP)

**Figure 13:**
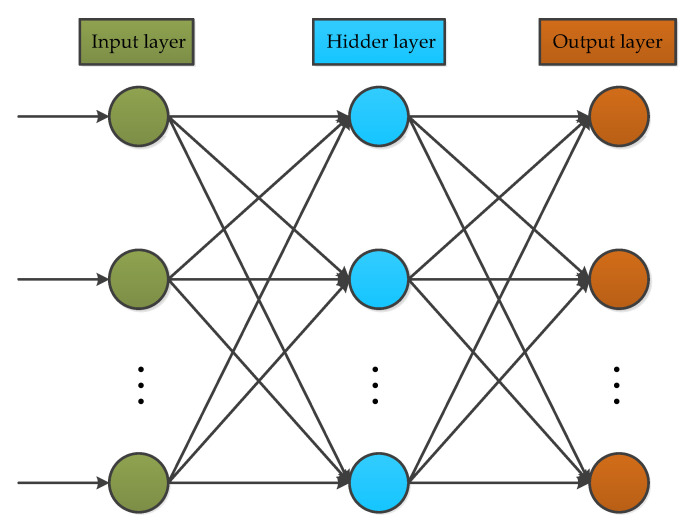
The classical architecture of Back propagation.

Back propagation neural networks (BP) are common neural networks, and are composed of three layers: an input layer, hidden layer, and output layer [56]. The classical structure is shown in Figure 13. The input layer nodes are used to choose independent variables for estimation. The hidden layer is an important part of the network, where the learning ability is tuned for better utilization and performance of the network. The output layer is employed to estimate the results against independent input variables. 

BP is successfully used in many applications, particularly for the MEMS gyroscope. For example, in references [57,58], BP is employed for modeling random drift and temperature compensation in a MEMS gyroscope, and the results obtained show that BP can yield better and accurate temperature compensation.

#### 3.5.2. Radial Basis Function Neural Network (RBF)

**Figure 14:**
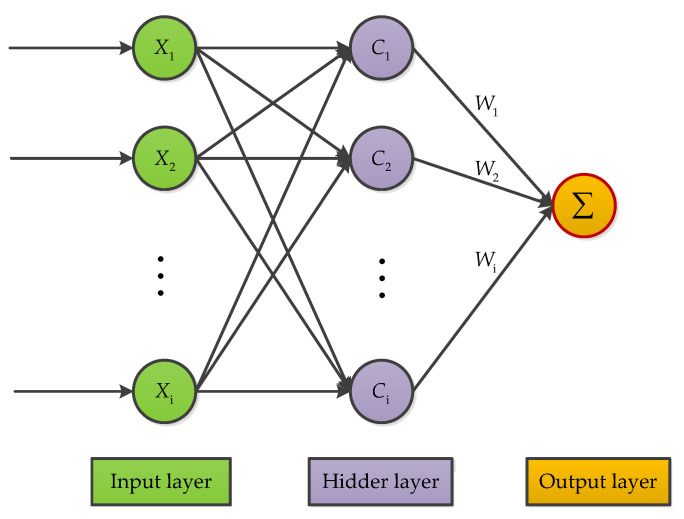
Radial basis function neural network structure.

The RBF is an artificial neural network that uses radial basis functions as activation functions; this makes it somewhat different from the BP neural networks. Although, both network approaches are commonly designed as a three-layered architecture. While BP is a global approximation to nonlinear mapping, RBF neural network is a local approximation. Therefore, the training speed and convergence speed of the RBF neural network are faster than BP [48]. As shown in Figure 14, the RBF neural network is a three-layer neural network, which includes an input layer, hidden layer, and output layer. The transformation from input space to hidden space is nonlinear, while the transformation from hidden space to output space is linear. Recently, this approach has been progressively stepping into the MEMS inertial sensors field. For instance, in reference [59,60], RBF models were developed and implemented for temperature compensation in the area of MEMS inertial sensors. The experimental results from the studies proved the method would make big progress in compensating temperature drift of MEMS gyroscopes and MEMS accelerometers.

#### 3.5.3. Support Vector Machine (SVM)

**Figure 15:**
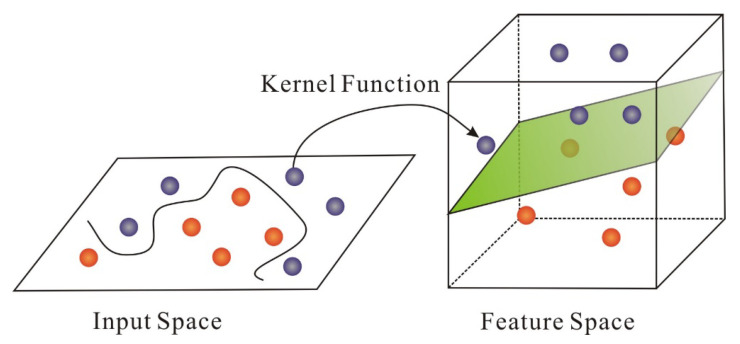
The key process of Support vector machine [63].

The SVM algorithm is a two-classification algorithm that classifies samples by constructing a hyperplane function [61]. SVM is mainly divided into linear SVM and nonlinear SVM [62]. Linear SVM is based on the Euclidean distance between samples to determine the structure of the division. Nonlinear SVM replaces the inner product with the convolution kernel function, which is equivalent to defining a generalized distance, and the generalized distance is used as the division basis [62]. The key of SVM lies in the kernel function. As showed in Figure 15, as long as the appropriate kernel function is selected, the classification function of the high-dimensional space can be obtained [63]. The SVM can solve linear and nonlinear problems and is applicable to many practical problems. In the MEMS inertial sensors field, the SVM can be used to establish error modeling and compensate the random drift. In addition, some existing experimental results have proved that the SVM model has shown vast improvement for error reduction, as well as high precision and good generalization ability [64,65].

#### 3.5.4. Relevance Vector Machine (RVM)

The relevance vector machine (RVM) was proposed by Tipping on the basis of the Bayesian framework in 2001 [66]. It has the same function form as the SVM. Like SVM, it converts the nonlinear problem of low-dimensional space into high-dimensional space based on the kernel function linear problem. The principle of RVM can be simplified as the following steps [67]: Firstly, select the appropriate kernel function, map the eigenvectors to the high-dimensional space, and use several common kernel functions. Then, initialize the super parameters α and variance $\sigma^{2}$. In RVM, α and $\sigma^{2}$ are solved iteratively, so it needs to be initialized. After that, solve the optimal weight distribution iteratively. Finally, anticipate new data. In recent years, the RVM has been used to compensate the random drift of MEMS gyroscope, and its performance was validated by experiments [67].

### 3.6. Deep Learning

#### 3.6.1. Wiener-Type Recurrent Neural Network (WRNN)

**Figure 16:**
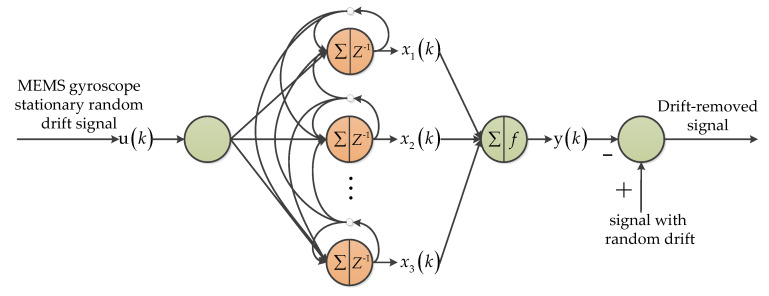
The random drift modeling and compensation process through the Wiener-type recurrent neural network [69].

The whole Wiener-type recurrent neural network (WRNN) can be divided into two main parts: a dynamic linear model and static nonlinear model. Figure 16 shows the principle of the recursive structure [68,69]. The input layer transmits the input values to the neurons of the dynamic layer. To infer the current state of the network, the dynamic layer integrates the current input information from the input layer with the state history stored in the memory of the dynamic layer neurons. The neurons in the output layer perform nonlinear transformations on state variables with different weights. To prove the feasibility of the WRNN algorithm, these papers developed the drift modeling and compensation algorithm, which is based on the WRNN to model the intrinsic drift of the gyroscopes [68,69].

#### 3.6.2. Neural Architecture Search Recurrent Neural Network (NAS-RNN)

**Figure 17:**
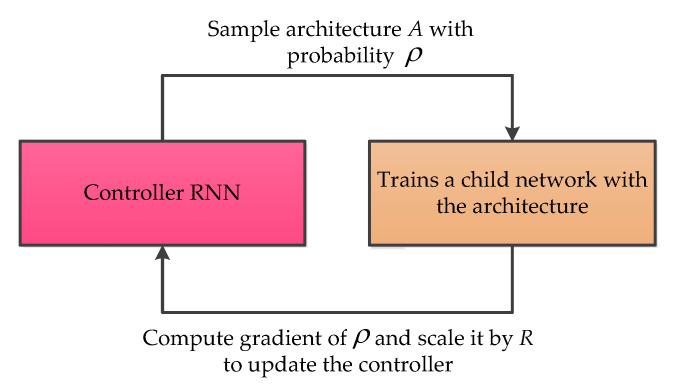
The basic architecture of neural architecture search recurrent neural network [70].

The neural architecture search recurrent neural network (NAS-RNN) was proposed by Barret Zoph in 2016 with the main purpose of using reinforcement learning to find an optimal network, while solving a problem [70]. Indeed, NAS-RNN was invented to solve time series problems in the data science community. Different from the conventional method, NAS-RNN was able to search a more feasible architecture for the selected application. Recently, this method was employed in MEMS gyroscope noise suppressing, and achieves considerable improvement for the MEMS gyroscope output signal [71]. The basic architecture of NAS-RNN is presented as follows in Figure 17.

#### 3.6.3. Long Short Term Memory (LSTM)

**Figure 18:**
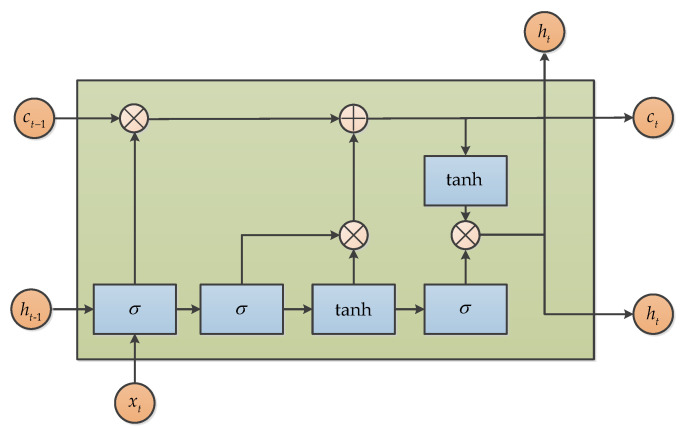
The basic structure of long short term memory.

Long short term memory (LSTM) is a popular variant of the Recurrent Neural Network (RNN), proposed in 1997 by Hochreiter [72], and also a kind of time cycle neural network, which is specially designed to solve the long-term dependence problem of common RNN, especially for MEMS gyroscope raw signal denoising [73,74]. The key idea of LSTM is the “three gates”, which are used to interact with cellular states and change the information carried by cellular states. LSTM uses two gates to control the contents of c in the unit state. One is the forgetting gate, which determines how much $c_{t}$ of the unit state at the last moment is retained until the current moment. The other is the input gate, which determines how much of the network’s input $x_{t}$ is saved to the cell state $c_{t}$ at the current moment. Lastly, the output gate is used to control how much of the unit state $c_{t}$ is output to the current output value $h_{t}$ of the LSTM. The basic structure of LSTM is showed as follows in Figure 18.

#### 3.6.4. Gate Recurrent Unit (GRU)

**Figure 19:**
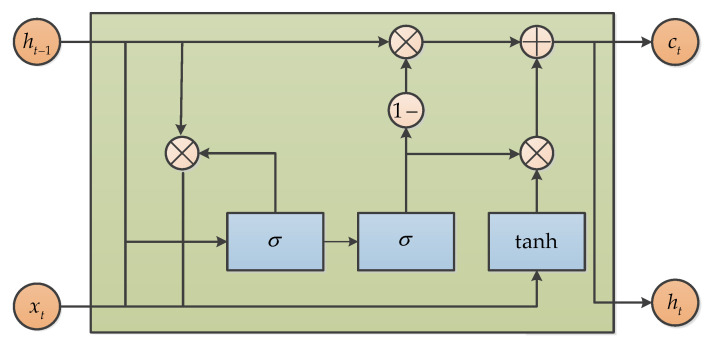
The structure of Gated Recurrent Unit.

Gated Recurrent Unit (GRU), which was proposed by Chung in 2014, is also a variance of LSTM [75]. It combines the forget gate and the input gate into a single update gate, so it has only three gates. It also mixes cell state and hidden state, and other changes. The final model is simpler than the standard LSTM model, and it is also a very popular variant. The structure of GRU can be expressed as in Figure 19. GRU was also used to model the raw signal and suppress noise by Jiang, and the results show that GRU can be effective for output signal denoising [74].

#### 3.6.5. Simple Recurrent Unit (SRU)

**Figure 20:**
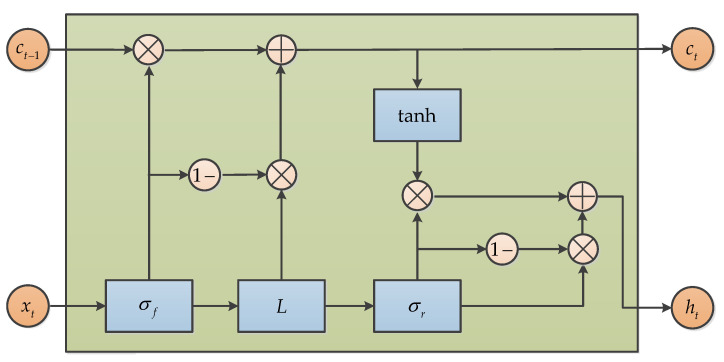
The structure of Simple Recurrent Unit.

Simple Recurrent Unit (SRU), which is a new variant of RNN based on LSTM and GRU research, was proposed by Tao Lei in 2018, and the SRU has a more succinct structure for accelerating the training procedure. When compared with LSTM and GRU, the SRU has a faster training speed that is derived from its unique structure, and there is no loss of accuracy, under the premise of ensuring training speed [76]. The basic structure of SRU is presented in Figure 20. Similarly, the SRU is adopted for MEMS gyroscope raw signals denoising and obtains good accuracy improvement [77].

### 3.7. Adaptive-Based Algorithms

#### 3.7.1. Recursive Least Squares (RLS)

The recursive least squares (RLS) is an adaptive filter, and it uses an iterative algorithm instead of matrix inversion to reduce the amount of calculation [78]. The basic idea of RLS is that the new estimated value is modified based on the old estimated value. For simple analysis, Θ is set as a vector, and Θ is only related to the current observation value. Then, the recursive least square method can be expressed as follows. RLS is usually used for signal filtering; in the MEMS inertial sensor field, RLS is adopted for estimating the stochastic error model of MEMS inertial sensor [78,79]. $y_{k}$ is output measurement; $\phi_{k}^{T}$ is input measurement.

(20) $${\overset{\wedge }{\Theta }}_{k}=\overset{\wedge }{\Theta_{k-1}}+K_{s}\varepsilon_{k}$$

(21) $$K_{k}=P_{k}\phi_{k}$$

(22) $$\varepsilon_{k}=y_{k}-\phi_{k}^{T}{\overset{\wedge }{\Theta }}_{k-1}$$

(23) $$P_{k}=P_{k-1}-\frac{P_{k-1}\phi_{k}\phi_{k}^{T}P_{k-1}}{1+\phi_{k}^{T}P_{k-1}\phi_{k}}$$

#### 3.7.2. Least Mean Squares (LMS)

The least mean squares (LMS) algorithm is a widely used adaptive filtering algorithm. This algorithm does not need to know the statistical characteristics of the input signal and the expected signal. It has the advantages of a simple principle, few parameters, fast convergence speed, and easy implementation. As showed in paper [80], the author designs an LMS filter in the MEMS gyroscope control system for improving the precision. LMS algorithm is based on Equations (24)–(27), where x(k) is the input signal, y(k) is the output of the filter, d(k) is the reference signal (desired signal), e(k) is the error signal, w(k) is the weight vector, μ is the iteration step-size, and $\lambda_{\max}$ is the maximum eigenvalue of auto correlation matrix of input signal.

(24) $$w(0),1<\mu <1/\lambda_{\max}$$

(25) $$y(k)=\sum_{i=0}^{N}w_{i}(k)x_{i}(k-i)$$

(26) e(k)=d(k)−y(k)

(27) w(k+1)=w(k)+2μe(k)x(k)

#### 3.7.3. Adaptive Sliding Mode Controller (ASMC)

**Figure 21:**
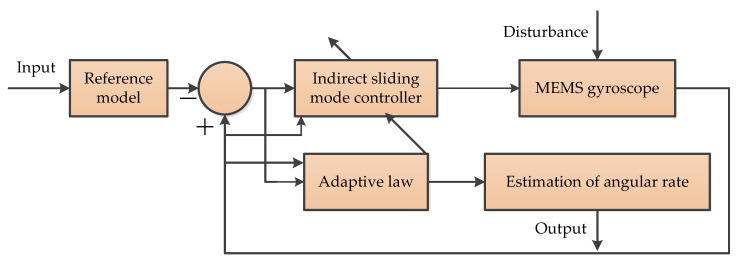
The principle of the adaptive sliding mode controller [82].

The adaptive sliding mode controller (ASMC) for the MEMS vibration z-axis gyroscope is employed in Fei’s research papers, which can estimate the angular velocity, damping coefficient, and stiffness coefficient in real time. The ASMC error compensation process was described in a study by Fei et al. [81]. The sliding mode compensator is used to reduce control chattering, while the adaptive law is used to update the parameters of the adaptive sliding mode controller. The simple principle diagram is shown in Figure 21, which presents an indirect adaptive sliding mode control for the MEMS gyroscope [82].

#### 3.7.4. Adaptive Kalman Filter (AKF)

**Figure 22:**
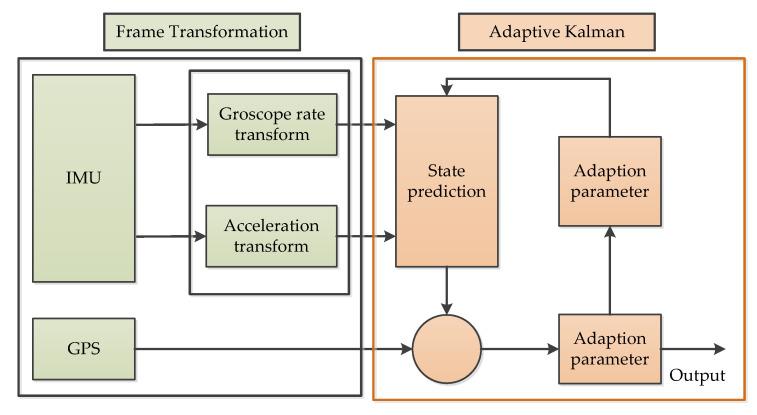
The system diagram of adaptive Kalman filtering [85].

Adaptive Kalman filtering (AKF) is mostly used for filtering measured data while constantly judging whether the system dynamics changed during the operation to give a real-time estimation and correction of model parameters, in cases of changes; and to adapt noise’s statistical characteristics to improve the filtering accuracy [78,83,84]. In reference [85], a model of AKF was developed and implemented for real-time estimation of the error covariance matrix. This technique can be used to improve the integrity of inertial measurement unit (IMU) and Global Positioning System (GPS) navigation for land vehicle applications, and attempts to design an AKF that performs better than traditional KF when GPS is interrupted. The system diagram is as shown in Figure 22 [85].

#### 3.7.5. Adaptive Filtering Based on Dynamic Variance Model (AF-DVM)

**Figure 23:**
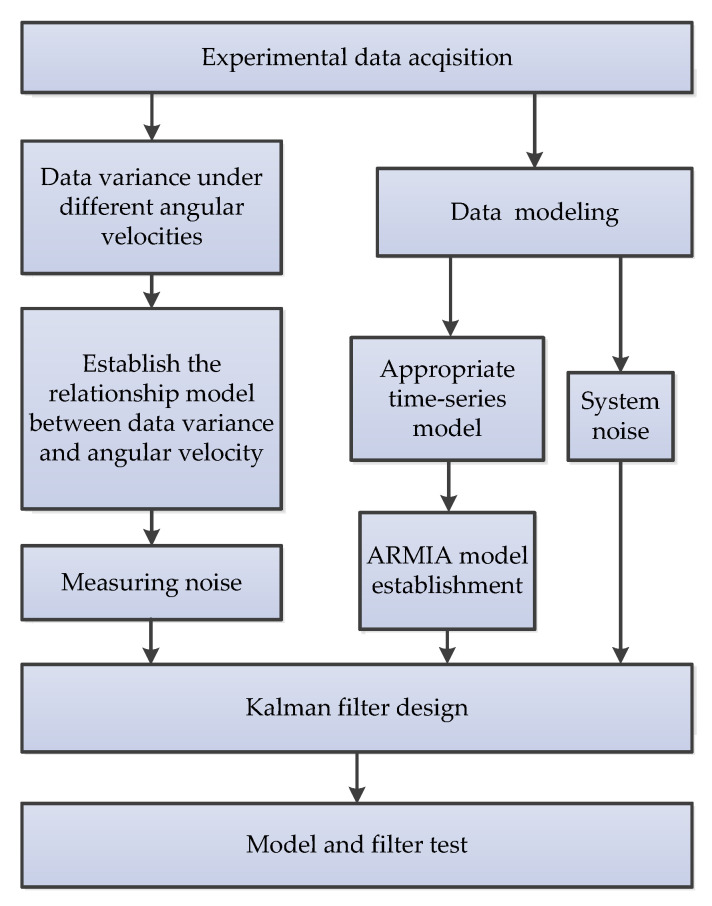
The adaptive filtering method based on the dynamic variance model [86].

An adaptive filtering method based on the dynamic variance model is introduced to compensate MEMS gyroscope random errors at different angular rates [86]. The principle is illustrated in Figure 23, which is derived from the original work in reference [86]. First, output data of different angular rate MEMS gyroscopes were collected, and the statistical characteristics of random errors of different angular rates were analyzed to establish the auto-regressive integrated moving average (ARIMA) model and dynamic variance model. After that, a KF is based on the ARIMA model. According to the dynamic model of data variance and angular rate, the method can adjust the KF noise coefficient online. Finally, the model and filter are verified by using the constant angular velocity and continuous variable angular velocity output data of the gyroscope, respectively.

### 3.8. Comparative Analysis of Existing Algorithms

For extensive comparative analyses of the existing algorithms proposed for improving accuracy in the MEMS inertial sensor, the corresponding algorithms in the reviewed articles were analyzed with respect to the seven groups discussed earlier. The details of their task, status of application for real-time and/or offline domains, working environment, and some observational remarks are presented in Table 1. In addition, a comparative study was carried out based on the structural characteristics, advantages/disadvantages of the algorithms, and strength of the methods in the application domains, as presented in Table 2. For readers’ clarity, the different groups each algorithm falls into were also compared in Table 3. For this purpose, algorithms in each group of the previous tables were combined, while the main tasks each group was commonly used for in the existing literature were summarized along with the advantages/disadvantages and the numbers of studies that the systematic comparison was based.

## 4. Discussion

An extensive search about precision improvement in MEMS technology, such as the inertial sensors, shows that despite the existing review papers for MEMS inertial sensors in domains like structure optimization in MEMS inertial sensors [8,21], developments in inertial sensitive structures [87,88], quality factor for tuning mechanisms [89], and corresponding interface circuits [8,90,91], there is no single review and overview of studies that focuses on processing MEMS inertial sensor output using signal-based algorithms to improve the sensor’s accuracy until now. Hence, this review article focuses on random error signal processing algorithms for improving the accuracy of MEMS inertial sensors and provides a detailed overview for users. Our main contribution in this review is the classification of a total of 30 algorithms filtered from 256 methods into seven categories for ease of comparison and evaluation. The proposed classes are simple filter algorithms, Kalman-based algorithms, wavelet-based algorithms, sensor fusion, machine learning, deep learning, and adaptive-based algorithms. Amongst the algorithms developed for improving the intrinsic accuracy of MEMS inertial sensors, Kalman-based algorithms are the most commonly used method for error compensation and noise suppression. Followed by these Kalman-based techniques are adaptive-based methods, which also showed some great potential in recent times. Next is the artificial intelligence algorithms; these can be adapted to solving error compensation and related problems in different application areas. Almost all the algorithms have potential to be implemented in simulation mode and within a hardware setup; furthermore, they can be used in their fundamental state, improved, and combined with other algorithms to improve the precision of the MEMS inertial sensor.

Simple filter algorithms such as fading memory filter, morphological filter, moving average filter, and variable bandwidth filters were applied to reduce raw data noise, static and dynamic noise, unstable periods drift, and low frequency vibration. Their mathematical principles are simple and easy to implement for real-time applications, whether in hardware or simulation. For preliminary signal processing, they are used alone or in combination. However, better results can usually be obtained if they are combined with other algorithms. Kalman-based algorithms are commonly applied for random drift compensation, error compensation, temperature drift compensation, damping and stiffness imperfections compensation, as well as improving the convergence of the KF. With a focus on random error signal processing algorithms, related Kalman-based algorithms, including the Kalman filter, extended Kalman filter, incremental Kalman filter, strong tracking Kalman filter, and discrete-time Kalman filter algorithms, are shown in this article. They are all based on the Kalman filter and mainly complete the signal denoising and random drift signal compensation for MEMS inertial sensors. Wavelet-based algorithms include wavelet threshold, improved wavelet threshold, adaptive stationary wavelet threshold, and EMD-based wavelet threshold. They can be applied for noise reduction, high frequency noise reduction, random drift or error compensation, and performance improvement. The wavelet-based algorithms provide a good solution when used to compensate random error or noise in inertial sensors under static and dynamic conditions. It usually provides good and reliable results, but sometimes, it needs to be combined with other algorithms to have a huge advantage.

Sensor fusion algorithms are the combination of data generated by homogeneous or heterogeneous sensors, so that the result information is more accurate and reliable when used with separate sensors. In this review, three methods, in which sensor fusion algorithms were used for noise suppression and drift/offset elimination, were discussed. This includes application in virtual gyroscope or gyroscope array, heterogeneous fusion, and combination sensors. In common cases, such sensors are all implemented in real time on MATLAB or DSP or FPGA, but they all need to add filtering algorithms in the last step to filter out unnecessary noise. Machine learning algorithms such as BP, RBF, SVM, and RVM have also been employed in MEMS inertial sensor output signal processing. This approach is commonly used for temperature compensation, random error modeling, and drift compensation. Generally, the methods produce real signals on the temperature control turntable, and then, perform signal processing offline. The results show that they can achieve good compensation effects and accuracy improvements. Deep learning in MEMS inertial sensor signal processing mainly refers to the application of recurrent neural networks and its variants. In particular, GRU, SRU, and WRNN are some other deep learning algorithms that were developed in the last five years but they have found a very large prospect for time series signal processing. In the domain of MEMS inertial sensor signal processing, recurrent neural network and its variants have shown good noise reduction effects; however, currently, they are mostly implemented in the Python 3.6 environment. Adaptive-based algorithms, such as RLS, LMS, ASMC, AKF, and AF-DVM, were used for dynamic estimation, random error compensation, the damping and stiffness coefficients estimation, and navigation precision improvement. Basically, almost all the mentioned adaptive algorithms are implemented for online processing of output (signal) from the MEMS inertial sensor and tailored for precision improvement. In essence, they usually achieve satisfactory performance and robustness. While a number of error compensation algorithms have been studied, it is vital to perform an overview on how these methods are properly selected and applied. Output signals from MEMS inertial sensors usually contain noise at varying frequency levels. As a correction measure, simple filtering and wavelet-based approaches are better used to deal with high frequency noise, while it is necessary to find other algorithms such as Neural Networks and SVM that can be applied to process noises that are in low frequency. In respective contexts, the inertial sensor output signal has high-dimensional and highly nonlinear characteristics; thus, learning-based methods, including algorithms based on neural networks and SVM, have shown some comparative advantages over the traditional filtering algorithms. While the algorithms based on SVM can overcome over-fitting and better generalization ability for small samples, those based on neural networks are prone to overfitting and limited generalization capabilities. The Deep Learning algorithms developed quickly in recent years have the advantage of large sample learning and prediction in the field of time series data processing, and further research is needed to realize the real-time high-precision estimation and compensation of random errors of inertial sensors. Kalman usually needs to combine the autoregressive moving average model (ARMA) to realize real-time suppression or compensation of inertial sensor drift error and random noise. ARMA usually assumes that random errors are linear combinations, and Kalman usually assumes that the state space and noise characteristics are Gaussian distributions. In practical applications, these noise models are not time-invariant. Therefore, the fixed variance of the process noise and measurement noise covariance matrix is not suitable for real-time applications, because it can lead to filter divergence of estimation. In order to solve the divergence problem, various adaptive algorithms or improved adaptive Kalman algorithms play an important role in practical applications due to their lower computing cost and real-time advantages. In addition, the sensor fusion algorithms are usually a combination of multiple sensors, but they also need to cooperate with the filtering algorithm to achieve high accuracy.

## 5. Conclusions

This paper mainly introduces an algorithm focused on random error reduction and accuracy improvement in MEMS inertial sensors. Forty-nine random error processing papers that focused on improving the precision of MEMS inertial sensors and that were published in the last ten years were selected and analyzed. The algorithms mainly include representative methods and related classification algorithms in the domain. Thirty mainstream algorithms were filtered from the papers and categorized into seven main groups. Each group was carefully investigated and summarized in terms of tasks that they were used in, as well as analysis on if the applications were for online or offline in real-time approaches, and the working environment where they were analyzed. Lastly, we made some vital remarks and highlighted their advantages and disadvantages.

The algorithms studied in this review are of great significance to the refinement of MEMS inertial sensors. This review aims to provide a guide for users studying random error reduction algorithms in the MEMS inertial sensor, as well as according to science and technology developmental trends [92,93], and some hotspots in the research field [59,63,94,95,96]. We also concluded on the following points to better drive the prospects of the algorithms for processing and suppression of random error in MEMS inertial sensors; viz., (1) choosing or improving the appropriate compensation algorithm would depend on accuracy requirements and application scenarios; (2) combination of common algorithms or sensor fusion could improve performance; (3) Smart MEMS inertial sensors integrated with artificial intelligence algorithms could provide better precision of the MEMS inertia sensor. At the same time, we also summarize the development of other related technologies of MEMS inertial sensors.

In fact, the most fundamental error for MEMS inertial sensors is still derived from the sensitive structure in micron scale, so it is necessary to explore the sensitive mechanism clearly, and then, optimize the MEMS sensitive device. The MEMS inertial device is mainly composed of a basic beam, spring, and mass block. The MEMS inertial device is easily influenced by temperature, rotation speed, attached mass, instant temperature field, material distribution, geometry, and dimension size [97,98,99,100,101,102], resulting in structure stress concentration, thermal stress, unstable resonant frequency, and other adverse phenomena. Therefore, in order to better design the MEMS/NEMS device, it is necessary to consider stress release, temperature insensitivity, geometric structure, scale effect, driving/detection mode, appropriate non-classical parameters, and rod model [97,98,99,100,101,102].

Another error source of the MEMS inertial sensor is that it is vulnerable to external disturbance in actual navigation application, as well as model uncertainties, orthogonal error, and mismatching of driving and detection modes, which all will affect the navigation accuracy. In order to improve the anti-interference and robustness of the MEMS, traditional methods such as proportional integral derivative (PID), fuzzy control, and sliding mode control are not enough to achieve good performance [103,104,105]. Therefore, it is necessary to consider some appropriate and advanced control algorithms to improve the robustness and high-precision tracking performance of the MEMS. Some scholars have proposed many advanced hybrid algorithms, such as adaptive fractional sliding mode control [105], super twisting PID sliding mode controller algorithm [104], adaptive sliding mode based algorithms [105], adaptive fuzzy sliding mode control [106,107,108], and neural learning based algorithms [109], were used for the control of a MEMS gyroscope, which has achieved good simulation results, and still need further optimization to be applied in the actual system.

For MEMS inertial sensor circuits, a high-precision, low-power, and low-noise capacitance detection amplifier circuit is needed to convert the capacitance change into other more convenient physical parameters for amplification and measurement. Therefore, the front-end detection and amplification circuit is the key part to determine the performance of the whole sensor. The designer needs to select the most appropriate topology according to the main design indicators, namely energy consumption, floor area, measurement time, and resolution. AD/DA conversion circuits are also important parts; especially, an analog to digital converter based on electromagnetism sigma delta modulators is the best choice, which can improve bandwidth, linearity, dynamic range, and full-scale range for MEMS internal sensors aiming at industrial grade and strategic grade applications [8,90]. In addition, the analog circuit has some shortcomings such as electronic noise, temperature drift, and self-calibration difficulty. Compared with the analog circuit, the digital drive and detection circuit can be implemented on one chip by DSP or FPGA, while the analog drive and detection circuit can only be implemented on different devices [8,90]. With the interface circuit, sigma delta conversion circuit, and digital chip technology design and implementation becoming more convenient and mature, the precision of MEMS inertial sensors will be further improved. For MEMS packaging technology, with the rapid development of MEMS packaging, wafer level and 3D integration are becoming more and more important [91]. At the same time, according to the development characteristics of packaging technology and the actual market application requirements, a variety of hybrid packaging forms will emerge as the times require. In the case of controllable cost, the current single sample packaging will gradually transition to the system in a package (SIP) level to achieve smaller volume, smaller power consumption, and more integrated output of functional signals, so as to improve the cost performance of products and meet the application requirements in special fields [91]. In short, the integration of MEMS and IC and heterogeneous integration with other sensors will be inevitable trends.

In MEMS inertial sensor applications, the application of MEMS inertial sensors in the field of consumer electronics is undoubtedly the largest market, but at present, inertial sensors have been applied in the fields of wheeled mobile platform navigation, mobile omniwheel robot trajectory tracking, industrial robots, hexacopter navigation control, wearable devices motion monitoring, underwater vehicle navigation, and torpedo and rocket navigation [110,111,112,113,114,115,116,117,118,119,120,121,122]. With the improvement of the precision of MEMS inertial sensors and the increase in industrial market demand, the demand for MEMS inertial sensors in the above fields will increase day by day. At the same time, it will be expanded to some special fields, such as earthquake monitoring and housing health monitoring.

## Figures and Tables

**Table 1:** Comparative analysis of existing algorithms with references status of task analysis and working approach.

Algorithm	# of Papers	Task Analysis	Real-Time and Online/Offline	Working Environment	Remark
FMF	1	Raw data noise reduction [33]	Online	AMD-Quadcore FX-8800pCPU platform	The results show that the proposed filter can effectively reduce the sensor’s noise.
MF	2	Noise suppression in the MEMS gyroscope [36]; MEMS gyroscope output signal denoising [37]	Real time	MATLAB	The simulation is better achieved in the static state and dynamic state; the principle is simple and has much less calculation in real time.
MAF	1	Suppress the signal’s unstable periods [38]	Collect data online and process the data offline	NA	Single and multiple rate dynamic experiment analysis, and synthetic signal denoising analysis.
VBF	1	Reduce the low frequency vibration and sensor noise [39]	Real time	MATLAB	Adaptive bandwidth filter provides smooth data in harsh environments and eliminates the low frequency vibration effects (<10 Hz).
KF	3	Random drift compensation [41]; Temperature drift compensation [42,44]	Offline	MATLAB	The proposed method can effectively reduce random drift and temperature drift not only on the conditions but also at constant rates.
EKF	1	Damping and stiffness imperfections compensation [43]	Offline	MATLAB and DSP	Numeric simulation and experiment EKF show consistent results.
IKF	1	To reduce large errors and improve the convergence of the KF [44]	Offline	MATLAB	Comparison of KF/AKF/AIKF
STKF	2	To compensate the temperature drift [42]; Error compensation and accuracy improvement [45]	Real time	DSP	Static and dynamic experiments; the algorithm is easily implemented; the measurement noise of the MEMS gyroscope in static and dynamic states can be reduced by 93.6% and 63.9%, respectively.
DTKF	1	Bias drift and noise reduction [46]	Real time	DSP	The greatest feature is the direct modeling for true angular rate to obtain an optimal estimate.
WT	2	Large noise reduction for low-precision MEMS gyroscope [47,50]	Real time	DSP	A large number of the constant and dynamic rates experiments were tested.
IWT	2	Error compensation [48]; High frequency noise reduction and random drift suppression [49]	Offline	NA	Experimental results indicate that the improved wavelet threshold is effective.
ASWT	1	High frequency noise restraint [50]	Real time	DSP	Experimental results show that the adaptive stationary wavelet threshold is better than traditional wavelet threshold denoising methods.
EMD-WT	1	To improve the performance of the high-G MEMS accelerometer [51]	Offline	NA	Experiment and verification in the Hopkinson Bar calibration system, and it decreases the noise of the original signal by 96%.
VG	2	To reduce the noise and improve the accuracy of the individual gyroscope [52,53]	Online	MATLAB/Simulink	Dynamic simulations and experiments with a six-gyroscope array were carried out.
HF	1	Real time calibration and long-term drift compensation [54]	Real time/Online	MATLAB	Intelligent Real-Time MEMS Sensor Fusion and Calibration.
CS	1	To eliminate the drift and offset [55]	Real time	DSP and FRGA	Various simulation and experimental results are presented demonstrating its effectiveness.
BP	2	Null drift, temperature compensation [57]; Compensation of temperature and acceleration effects [58]	Real time	NA	Bias instability shows 57% improvement; Temperature test from −40 to −80 °C; BP NN yields accurate temperature compensation.
RBF	3	Random error compensating [48]; Temperature compensation [60,63]	Real time [60] Offline [48,63]	NA	Good generalization ability, higher precision prediction, and compensation ability; A new fusion algorithm is proposed and proved in temperature test equipment.
SVM	3	Modeling and compensation [63,65]; Error modeling [64]	Offline	MATLAB/LibSVM	SVM has high precision and good generalization ability; thus, experimental results proved that the SVM approach reduced the noise standard deviation by 10–35% for gyroscopes and 61–76% for accelerometers.
RVM	1	Random drift compensation [67]	Offline	NA	Static and dynamic experiments were conducted.
WRNN	2	Random drift modeling and compensation [68,69]	Real time	MCU	The effectiveness of the proposed WRNN-based random drift modeling and compensation scheme for the MEMS-based gyroscopes was successfully validated.
NAS-RNN	1	Noise suppressing [71]	Offline	NA	The NAS-RNN was effective for MEMS gyroscope noise suppressing.
GRU	1	Noise suppressing [74]	Offline	Python	The mixed deep recurrent neural networks outperformed GRU-GRU and LSTM-LSTM.
SRU	1	Signal denoising [77]	Offline	Python	The results surely demonstrated the effectiveness of the employed SRU in this application.
RLS	2	Random noise reduction [78]; Online dynamic estimation of inertial sensor error model [79];	Online	STM32 microcontroller [78]; DSP [79]	The results show that RLS can effectively reduce the prediction error compared with non-recursive estimation.
LMS	1	Signal error processing [80]	Online	DSP Builder/FPGA	The results show that it is reliable and has high precision.
ASMC	2	Estimate the angular velocity and the damping and stiffness coefficients [81,82]	Offline	MATLAB/Simulink	It has satisfactory performance and robustness in the presence of model uncertainty and external disturbance.
AKF	4	Noise reduction [78]; Static and dynamic noise reduction [83]; The drift error and random noise restraint [84]; Navigation precision improvement [85]	Real time [78,84,85]	STM32 microcontroller [78]; DSP [84,85]	It is shown that AKF has a better performance rather than conventional KF.
AF-DVM	1	Dynamic random error compensation [86]	Online	DSP	The proposed method was verified through a constant angular rate and continuous variable angular rate turntable experiments.

**Table 2:** Comparative analysis of individual algorithms and their merits.

Group	Algorithms	Structure Characteristics	Advantages	Disadvantages	Strength in Application Domain
Simple filter algorithms	FMF	The FMF structure is very similar to KF	Very low computational overhead and KF divergence suppression	The optimal filter gain is not easy to find	To reduce the sensor’s noise and track moving objects in radar applications and medical devices
	MF	Four basic operators as follows: dilation, erosion, opening, and closing	It is simple, fast, and real-time	MF generally suffers from different output biases and the scale selection problems of structural elements	In order to filter out the noise of the MEMS gyroscope in vehicle mobile satellite communication
	MAF	MAF is the first choice of time domain signal, and is the most common in DSP	Fast convergence rate and small steady-state errors	MAF shows certain lag	It is applicable for signal denoising under arbitrary motion state conditions
	VBF	VBF processes data by sinusoidal data estimation	It can be implemented real-time	As the bandwidth decreases, the time delay increases	Real flight conditions
	KF	Filter computation loop and gain computation loop	Small amount of calculation	It can only fit linear Gaussian systems.	Sensor data fusion
	EKF	EKF is a kind of pseudo nonlinear KF	Small and fast calculations	Less effective for highly nonlinear problems and poor robustness	Unmanned aerial vehicles
Kalman-based algorithms	IKF	IKF also is a nonlinear KF	Better estimation accuracy and more robust to the unstable system	It has a larger calculation amount, but still can satisfy the real-time requirement.	In the airborne strapdown inertial navigation system application
	STKF	Nonlinear adaptive filter	Strong robustness	The sequence of residuals should be orthogonal at all times	With potential to be used in adaptive control of flexible robot
	DTKF	A type of optimal KF	Direct modeling for angular rate signal	The filtered rate signal has an auto-correlation	Aviation and aerospace navigation
	WT	Hard threshold and soft threshold	No need to establish accurate error model; Fast computation, and broad adaptability	The Pseudo-Gibbs will appear at the discontinuity of the signal	Primarily applicable in the case of white noise in the signal processing
Wavelet-based algorithms	IWT	In addition to soft and hard threshold function, a new threshold function is added	Better adaptability	It is very difficult to find an ideal threshold	Indoor inertial navigation systems
	ASWT	Redundancy, translation-invariance, and more approximate estimation	Time invariance; simple and more smoothing	The computation load will increase	Application in the case of dynamic signal with high frequency noise restraining
	EMD-WT	Combination of two algorithms	Suitable for nonlinear and non-stationary signals; Faster, more reliable, and efficient than single methods	It is quite difficult to remove noise in real time	Monitoring natural disasters and various navigation control
Sensor fusion algorithms	VG	Gyroscope array	Accuracy of virtual gyro is higher than single gyro	It still needs KF filter	Navigation and guidance
	HF	Fusion of gyroscopes, accelerometers, and magnetometers	Faster dynamic response; Converges faster and take less computational time	Higher CPU load	Attitude and heading reference systems
	CS	Combines rotary encoders and gyroscopes; Low computational demands and negligible parameter tuning effort	A viable alternative to high-resolution encoders;	It still needs to further restrain the disturbance	Servo motors or robot joints
	BP	Input layer, hidden layer, and output layer	Nonlinear function relationship model	Time-consuming and its denoising accuracy depends on personal experience	To effectively improve the accuracy and practicability of flight attitude angle calculation
Machine Learning	RBF	Input layer, hidden layer, and output layer	The training speed and convergence speed of the RBF are faster than BP	Need to combine with other algorithms for high accuracy	High-G MEMS accelerometer temperature compensation; Application in navigation, defense, and impact measurement.
	SVM	It is a two-classification algorithm that classifies samples by constructing a hyperplane function	Better generalization ability for small samples	It is difficult to learn and predict large samples	North-seeking, navigation, pedestrian step estimation, pattern recognition, and many other fields
	RVM	It is a sparse probability model	The generalization ability of RVM is better than SVM	The training time is a little long	Guidance, navigation, and control systems for space vehicles
	WRNN	A dynamic linear model cascaded by a static nonlinear model	The algorithm is integrated into the real application	It still needs to integrate a lowpass filter	Handwriting Trajectory Reconstruction
Deep Learning	NAS-RNN	Neural networks with reinforcement learning	The NAS-RNN superiority compared with the LSTM-RNN	More heavy computation load	Various vehicles, carriers, and smart devices
	LSTM	A type of RNN	LSTM performs better in longer sequences	More parameters and more difficult training	Image processing, nature language processing, and sequential signal processing
	GRU	A type of RNN	GRU is much easier to train than LSTM and can greatly improve training efficiency	GRU parameters are fewer and therefore, easier to converge, but LSTM expression performance is better for large datasets	Image processing, nature language processing, and sequential signal processing
	SRU	A new type of RNN based on LSTM and GRU	The SRU has faster training speed than LSTM and GRU	It still needs further research	Image processing, nature language processing and sequential signal processing
	RLS	A type of adaptive filter	Convergence speed is very fast	Different inertial sensors need different forgetting factor	Automobile industry, flight vehicle, and robotics
	LMS	A widely used type of adaptive filter	Simple principle, few parameters, fast convergence speed and easy implementation	Need to combine other algorithms for good performance	It can be integrated into the FPGA for various real applications.
Adaptive-based algorithms	ASMC	Sliding mode controller	More high robustness	The simulations are only performed	Environment variations and external disturbances from the real system
	AKF	NA	AKF performs better than traditional KF	NA	Land vehicle applications
	AF-DVM	Algorithm combination	Adaptive dynamic random error compensation is validated	NA	Inertial measurement and inertial stabilization

**Table 3:** Comparative analysis of algorithms based on group classification and their merits.

Group	Main Tasks	Advantages	Disadvantages	Number of Studies
Simple filter algorithms	Raw data noise reduction Noise suppression in MEMS gyroscope Suppress the signal’s unstable periods Reduce the low frequency vibration and sensor noise	It has a simpler construction Low computational overhead It is easy to be implemented in real time	Not easy to find the optimal filter gain Has a statistical bias problem Not simple to choose an optimal length for the SE Need to work together with other algorithms for better noise reduction	5
Kalman-based algorithms	Random drift compensation Temperature drift compensation Damping and stiffness imperfections Compensation Error compensation and accuracy improvement Bias drift and noise reduction	One of the most common signal processing algorithms for MEMS inertial sensors Can be easily implemented on MATLAB and DSP Limited computation capacity Can effectively reduce the static and dynamic error	Sometimes, it needs to combine other algorithms for noise reduction Once, subsystem faults greatly affect the compensation precision If the environment changes drastically, the drift model is difficult to maintain high accuracy	8
Wavelet-based algorithms	Large noise reduction for low-precision MEMS gyroscope Random error compensation High frequency noise reduction and random drift suppression To improve the performance of high-G MEMS accelerometer	Can effectively reduce the static and dynamic error Multi-resolution analysis in time domain and frequency domain simultaneously Simple algorithm and small computational complexity No need to establish the system error model	Sometimes, it needs to work together with other algorithms Poor adaptability Difficult to find ideal wavelet threshold	6
Sensor fusion algorithms	To reduce the noise and improve the accuracy of the individual gyroscope Real time calibration and long-term drift compensation To eliminate the drift and offset	Can be implemented in real time Less computational time ∙	Large volume and integrated error It must combine other filter algorithms Sometimes, it needs information from other sensors, e.g., MEMS accelerometer or magnetometer or rotary encoders	4
Machine Learning	Compensation of temperature and acceleration effects Error compensation Temperature compensation Error Modeling and compensation	One of the most common signal processing algorithms for MEMS inertial sensors It is easy for small training data The network structure is relatively simple SVM needs less training time	More computational time Not easy to implement for large-scale training data NN is easily over-fitting Sometimes, it needs to work with other algorithms	9
Deep Learning	Random drift modeling and compensation Noise suppressing Signal denoising	One of the most common signal processing algorithms for MEMS gyroscope in recent three years Focusing on time series signal prediction processing	More computational time It is relatively difficult for real time signal processing The network structure is relatively complexity Limited data were trained in those papers	7
Adaptive-based algorithms	Random noise reduction Dynamic estimation of inertial sensor error mode Estimate the angular velocity and the damping and stiffness coefficients Static and dynamic noise reduction Navigation precision improvement	The most common signal processing algorithms Almost all can be implemented in real time Easily be implemented in hardware	Sometimes, it needs to work together with other algorithms Need to enhance the practicability	10
